# Current status, trend changes, and future predictions of the disease burden of type 1 diabetes kidney disease in global and China

**DOI:** 10.3389/fendo.2025.1559363

**Published:** 2025-03-17

**Authors:** Qinghua Yang, Li Jin, Mingwei Luo, Shiwei Xie

**Affiliations:** ^1^ Department of Endocrinology, Panzhihua Central Hospital, Panzhihua, China; ^2^ Department of Medical Records Statistics, Panzhihua Central Hospital, Panzhihua, China; ^3^ Department of Orthopedics, Panzhihua Central Hospital, Panzhihua, China

**Keywords:** ARIMA prediction, chronic kidney disease, global disease burden, incidence, mortality, type 1 diabetes, trend analysis, years lived with disability

## Abstract

**Objective:**

This study analyzes the global and China trends in the prevalence, disease burden, and future projections of Type 1 Diabetic Nephropathy (T1DN) over the past three decades, providing data to inform public health policies and clinical interventions.

**Methods:**

Data from the Global Burden of Disease (GBD) 2021 database were used to analyze the incidence, prevalence, mortality, years lived with disability (YLDs), years of life lost (YLLs), and disability-adjusted life years (DALYs) of T1DN globally and in China from 1990 to 2021. Trend analysis was conducted using R and Joinpoint software, and the ARIMA model was applied to predict future trends in T1DN prevalence for the next 20 years. A significance level of p<0.05 was applied.

**Results:**

Globally, deaths from T1DN increased from 49,300(95% CI: 39,088-61,207) in 1990 to 94,020 (95% CI: 71,456-119,984)in 2021, with the age-standardized mortality rate remaining stable. DALYs rose from 2,227,518(95% CI: 1,835,372-2,679,207) in 1990 to 3,875,628 (95% CI: 3,062,395-4,845,503) in 2021, though the age-standardized rate slightly decreased. In China, the mortality rate declined significantly, and DALYs decreased, with the age-standardized DALYs dropping from 80.915/100,000(95% CI: 65.121-98.391)to 47.953/100,000(95% CI: 36.9-60.734). Globally, both incidence and prevalence increased, with global incidence reaching 95,140(95% CI: 82,236-111,471) cases and prevalence rising to 6,295,711 (95% CI: 5,459,693-7,114,345)cases. In China, incidence showed a declining trend, but prevalence continued to rise. The ARIMA model forecasts global incidence will reach 115,000 cases, with prevalence reaching 7,000,000 by 2041. In China, incidence is expected to stabilize, while prevalence may increase to approximately 2,500,000 cases.

**Conclusion:**

The burden of T1DN is rising globally, especially in terms of prevalence, while China has made progress in reducing mortality and disease burden. However, challenges remain in chronic disease management. Over the next 20 years, global prevalence is projected to continue increasing, while China’s prevalence may stabilize. Targeted interventions for different age groups and genders will be essential in reducing the T1DN burden.

## 1 Introduction

Type 1 Diabetes (T1D) is an autoimmune disease characterized by the destruction of pancreatic β-cells, typically occurring in childhood or adolescence (1, 2). Unlike Type 2 Diabetes, T1D has a more complex etiology involving genetic susceptibility and environmental triggers (3–6). In recent years, the incidence of T1D has been steadily increasing globally, particularly in developed countries (7). Previous studies have indicated that the number of T1D patients worldwide is significantly increasing posing substantial challenges to patients’ quality of life, as well as to social healthcare resources and public health systems (8). As the burden of T1D grows, it places increasing strain on global healthcare infrastructure, emphasizing the need for public health interventions and resource allocation across a range of healthcare sectors. In China, although the incidence of T1D is relatively low, the absolute number of cases remains concerning due to the large population (9). With improvements in diagnostic technologies and the accumulation of health data, our understanding of the pathological mechanisms and incidence trends of T1D is gradually deepening, but precise predictive models and intervention strategies still need further exploration.

Type 1 Diabetic Nephropathy (T1DN) is one of the most severe complications of T1D and a leading cause of End-Stage Renal Disease (ESRD) (10–12). T1DN typically progresses through stages such as microalbuminuria, overt proteinuria, and declining renal function, eventually leading to kidney failure (13, 14). This disease not only significantly increases the mortality risk for patients but also has a profound impact on their quality of life. The economic burden of treating T1DN is considerable, both for individuals and healthcare systems, and addressing this challenge is critical for long-term public health planning. Globally, the incidence and related mortality rates of T1DN vary significantly due to regional differences and the accessibility of healthcare resources (15–17). As a developing country, China faces many challenges in managing T1D and T1DN, including delayed diagnoses, poor patient compliance, and high treatment costs. Currently, the treatment strategies for T1DN mainly focus on delaying disease progression, and there is still no curative method. Moreover, as the life expectancy of diabetic patients increases, the burden of T1DN will further escalate in the future, placing higher demands on the healthcare system. This underscores the need for greater investment in preventive healthcare, early screening, and effective management strategies for chronic diseases like T1DN, which will significantly impact population health in the coming decades.

Although the pathogenesis and risk factors of T1D and T1DN have been widely studied, there are still many unanswered questions in the epidemiological field. First, research on the burden of T1DN globally and regionally is hindered by uneven data distribution, particularly a lack of data from developing countries and remote areas. Second, most of the current predictions on the burden of T1DN rely on traditional models and fail to fully utilize multidimensional data and advanced analytical techniques, such as machine learning and big data analysis (18–21). More sophisticated data modeling approaches are essential to provide accurate predictions of future disease burden, which is critical for effective public health policy and resource allocation.Furthermore, existing studies tend to focus on specific regions or time points, lacking a comprehensive comparison of global and Chinese trends (9, 22). Therefore, this study aims to comprehensively analyze the epidemiological trends and disease burden of T1DN in both global and Chinese contexts, using data from the Global Burden of Disease (GBD) database and innovative predictive models. By applying these advanced techniques, we aim to provide actionable insights that will guide public health interventions and inform healthcare policy on both local and global levels. Through this research, we not only address the gaps in current studies but also provide scientific evidence for future policy-making and public health interventions, advancing the prevention and treatment of diabetic nephropathy toward a more precise and effective direction.

## 2 Materials and methods

### 2.1 Data sources

The data for this study were obtained from the GBD. GBD is the most comprehensive and detailed research on health levels and trends globally, providing a unique platform for comparing the severity of diseases, injuries, and risk factors across different age groups, sexes, countries, regions, and time periods (23). The GBD database offers policymakers, public health leaders, researchers, and the public an opportunity to compare health progress across countries, helping to identify major preventable health losses, such as those caused by hypertension, smoking, and household air pollution. The data used in this study were sourced from the GBD 2021 Public Database (https://ghdx.healthdata.org/). GBD 2021 provides data on incidence, prevalence, mortality, years lived with disability (YLDs), years of life lost (YLLs), disability-adjusted life years (DALYs), and their corresponding 95% uncertainty intervals (UI) for 369 diseases and injuries from 1990 to 2021. Data come from 86,249 sources, including censuses, household surveys, civil registration, disease registries, and other public health databases. Disease coding follows the International Classification of Diseases (ICD), and GBD employs rigorous standardization, processing, and modeling methodologies.

Variables obtained from the GBD database for this study include the incidence, prevalence, DALYs, YLDs, YLLs, and their respective age-standardized rates (ASRs) of T1DN for both global and Chinese populations. These data are stratified by age (0-14 years, 15-19 years, and every 5-year group up to 95+ years), calendar years (1990–2021), sex, and country. This study assessed the incidence, prevalence, and mortality of T1DN and calculated age-standardized rates, including age-standardized incidence rates (ASIR), age-standardized prevalence rates (ASPR), and YLDs. Additionally, crude incidence rates (CIR), crude prevalence rates (CPR), and crude YLDs (CYR) for each age group were calculated.

### 2.2 Statistical analysis

Statistical analysis and visualization were performed using R software (version 4.4.0) and Joinpoint software (version 5.1.0), with a significance level of p<0.05. The assessed indicators include the incidence, prevalence, YLDs, and DALYs of T1DN, as well as ASIR, ASPR, ASYR, and ASDR. These age-standardized indicators (ASIR, ASPR, ASYR, ASDR) were sourced from the GBD database for both global and Chinese populations. Additionally, CIR, CPR, CYR, and crude DALYs rates (CDR) for each age group were calculated. Joinpoint software (National Cancer Institute, Rockville, MD, USA) was used to calculate the annual percent change (AAPC) and corresponding 95% confidence intervals (95% CI) to determine trends in the disease burden. The Joinpoint regression method segments the data and fits piecewise linear models to control for potential confounding factors, identifying trend changes while adjusting for baseline variations in the data (24). The log transformation of age-standardized indicators was modeled using the regression formula: ln(y) = α + βx + ϵ, where y represents the age-standardized indicator, x is the calendar year, α and β are regression coefficients, and ϵ is the error term. This method assumes that the trends in the data are piecewise linear, and the model adjusts for potential confounders by segmenting the time series into different periods. The annual percent change (APC) is derived from this model using the formula: 100×(exp(β)-1). If the 95% confidence interval of APC is greater than 0, the age-standardized indicator is considered to be increasing; if less than 0, it is decreasing; and if the confidence interval includes 0, the indicator is considered s. This approach assumes that trends follow a linear pattern within each segment, and changes in trend are identified at joinpoints.

### 2.3 ARIMA forecasting

The Autoregressive Integrated Moving Average (ARIMA) model was used to forecast the trend of T1DN prevalence for the next 20 years (ARIMA(p, d, q): p = autoregressive order, d = differencing order, q = moving average order). Differencing was applied to make the non-stationary data stationary, and autocorrelation functions (ACF) and partial autocorrelation functions (PACF) were used for validation. The optimal ARIMA model was selected using the autableto.arima() function based on the Akaike Information Criterion (AIC) (25). The model assumes that the time series data are stationary after differencing and that the residuals are independent and identically distributed (white noise).The ARIMA model analyzes time series data and removes trend and seasonal effects to uncover potential long-term patterns while controlling for confounding factors. Residual analysis ensured a good fit of the model, including checking for normality through ggplot and evaluating the ACF/PACF to verify the model’s validity. The Ljung-Box test was performed to ensure that the residuals did not exhibit serial correlation (white noise). This model assumes that the future values are linearly dependent on past values, and the residuals should exhibit no autocorrelation. The forecast performance of the ARIMA model was assessed using the following metrics: mean error (ME), root mean square error (RMSE), mean absolute error (MAE), mean percentage error (MPE), mean absolute percentage error (MAPE), and mean absolute scaled error (MASE).These metrics provide a comprehensive evaluation of the model’s forecasting accuracy. This study used data from publicly available databases (GBD 2021), and thus does not involve direct human subjects, nor does it require ethical approval.

## 3 Results

### 3.1 Trends in the prevalence and disease burden of chronic kidney disease due to type 1 diabetes in China and globally from 1990 to 2021

The trends in the prevalence and disease burden of chronic kidney disease (CKD) due to type 1 diabetes exhibited distinct patterns in both global and Chinese populations from 1990 to 2021 (**Table 1**). Globally, the number of deaths increased from 49,300 cases in 1990 (95% CI: 39,088-61,208) to 94,020 cases in 2021 (95% CI: 71,457-119,984). However, the age-standardized death rate remained almost unchanged, at 1.08 per 100,000 in 1990 and 1.084 per 100,000 in 2021 (AAPC = -0.1887, 95% CI: -0.28195 to -0.0953,*p*=0.000075), suggesting that despite a rising number of deaths, global control efforts have not led to a significant reduction in the death rate. In contrast, in China, the number of deaths rose from 19,457 in 1990 (95% CI: 15,417-24,181) to 20,688 in 2021 (95% CI: 15,277-27,014), but the age-standardized death rate decreased significantly, from 1.8 per 100,000 to 1.058 per 100,000 (AAPC = -1.8437, 95% CI: -2.0026 to -1.6846,*p*=0), indicating the effectiveness of China’s control measures and improvements in healthcare.

**Table 1:** All-age cases and age-standardized incidence, prevalence, YLDs, LLYs and DALYs rates and corresponding AAPC of Chronic kidney disease due to diabetes mellitus type 1 in Global and China in 1990 and 2021.

Location	Measure	1990		2021		1990–2021 AAPC	*p* values
		All-ages cases	Age-standardized rates per 100,000 people	All-ages cases	Age-standardized rates per 100,000 people		
		n (95% CI)	n (95% CI)	n (95% CI)	n (95% CI)	n (95% CI)	
**Global**	Deaths	49300 (39088-61208)	1.08 (0.837-1.354)	94020 (71457-119984)	1.084 (0.829-1.38)	-0.1887 (-0.28195,-0.0953)	0.000075
	DALYs (Disability-Adjusted Life Years)	2227518 (1835373-2679208)	47.049 (38.4-57.315)	3875628 (3062396-4845503)	45.204 (36.008-56.346)	-0.1276 (-0.185,-0.0702)	0.000013
	YLDs (Years Lived with Disability)	130365 (92665-172844)	2.874 (2.036-3.822)	294936 (206539-393036)	3.413 (2.397-4.544)	0.5288 (0.2663,0.7919)	0.000003
	YLLs (Years of Life Lost)	2097154 (1699743-2551170)	44.175 (35.694-54.273)	3580692 (2826228-4518921)	41.791 (33.257-52.6)	-0.3503 (-0.4215,-0.2791)	0
	Prevalence	2967857 (2607069-3328285)	57.538 (50.867-64.109)	6295711 (5459693-7114345)	77.313 (66.913-87.584)	0.9557 (0.847,1.0645)	0
	Incidence	63601 (52476-76375)	1.098 (0.917-1.309)	95140 (82237-111471)	1.31 (1.118-1.548)	0.5779 (0.551,0.6048)	0
**China**	Deaths	19457 (15417-24181)	1.8 (1.417-2.255)	20688 (15277-27014)	1.058 (0.79-1.377)	-1.8437 (-2.0026,-1.6846)	0
	DALYs (Disability-Adjusted Life Years)	916754 (739349-1115671)	80.915 (65.121-98.391)	886025 (666773-1127677)	47.953 (36.9-60.734)	-1.569 (-1.8383,-1.5125)	0
	YLDs (Years Lived with Disability)	37327 (24503-51465)	3.64 (2.372-5.055)	94133 (61403-131967)	4.549 (3.004-6.254)	0.8037 (0.4677,1.1407)	0.000003
	YLLs (Years of Life Lost)	879426 (700619-1076146)	77.275 (61.884-94.913)	791891 (591272-1031085)	43.404 (33.05-55.969)	-1.9475 (-2.1372,-1.7574)	0
	Prevalence	249863 (208019-294978)	22.106 (18.47-26.04)	493149 (411923-583500)	28.317 (23.947-33.226)	0.7913 (0.6397, 0.9432)	0
	Incidence	9475 (4977-15577)	0.855 (0.451-1.405)	6321 (3949-9464)	0.702 (0.381-1.144)	-0.6249 (-0.7416,-0.5081)	0

Globally, the disease burden of CKD due to type 1 diabetes has continuously increased. In 1990, the Disability-Adjusted Life Years (DALYs) were 2,227,518 (95% CI: 1,835,373-2,679,208), rising to 3,875,628 in 2021 (95% CI: 3,062,396-4,845,503). Despite this increase, the age-standardized DALY rate slightly declined, from 47.049 per 100,000 to 45.204 per 100,000 (AAPC = -0.1276, 95% CI: -0.185 to -0.0702,*p*=0.000013), reflecting some global improvements in healthcare, though the overall disease burden remains high. In contrast, China experienced a decline in DALYs, from 916,754 in 1990 (95% CI: 739,349-1,115,671) to 886,025 in 2021 (95% CI: 666,773-1,127,677), and the age-standardized rate significantly decreased from 80.915 per 100,000 to 47.953 per 100,000 (AAPC = -1.569, 95% CI: -1.8383 to -1.5125,*p*=0), which points to China’s success in reducing both disease burden and mortality. This indicates that China has made substantial progress in reducing the disease burden of CKD.

The trend in prevalence has shown a similar upward trajectory in both global and Chinese populations. Globally, the number of cases increased from 2,967,857 in 1990 (95% CI: 2,607,069-3,328,285) to 6,295,711 in 2021 (95% CI: 5,459,693-7,114,345), and the age-standardized prevalence rose from 57.538 per 100,000 to 77.313 per 100,000 (AAPC = 0.9557, 95% CI: 0.847 to 1.0645,*p*=0). This increase reflects an aging population and more people living with the disease globally. In China, the prevalence grew from 249,863 cases in 1990 (95% CI: 208,019-294,978) to 493,149 cases in 2021 (95% CI: 411,923-583,500), with the age-standardized prevalence rising from 22.106 per 100,000 to 28.317 per 100,000 (AAPC = 0.7913, 95% CI: 0.6397 to 0.9432,*p*=0). This suggests that while the disease burden has increased in China, it has done so at a slower rate compared to the global population.

Although prevalence has been rising, the incidence trends differ. Globally, the incidence increased from 63,601 cases in 1990 (95% CI: 52,476-76,375) to 95,140 cases in 2021 (95% CI: 82,237-111,471), and the age-standardized incidence increased from 1.098 per 100,000 to 1.31 per 100,000 (AAPC = 0.5779, 95% CI: 0.551 to 0.6048,*p*=0). In contrast, China experienced a decline in incidence, from 9,475 cases in 1990 (95% CI: 4,977-15,577) to 6,321 cases in 2021 (95% CI: 3,949-9,464), and the age-standardized incidence decreased from 0.855 per 100,000 to 0.702 per 100,000 (AAPC = -0.6249, 95% CI: -0.7416 to -0.5081,*p*=0). This suggests that, while global incidence is still rising, China has made progress in reducing new cases through early detection and better disease management.

In terms of quality of life, both global and Chinese trends exhibit notable differences. Globally, YLDs due to CKD resulting from type 1 diabetes increased from 130,365 in 1990 (95% CI: 92,665-172,844) to 294,936 in 2021 (95% CI: 206,539-393,036), with the age-standardized rate rising from 2.874 per 100,000 to 3.413 per 100,000 (AAPC = 0.5288, 95% CI: 0.2663 to 0.7919,*p*=0.000003). This indicates a growing burden on the quality of life globally. Years of Life Lost (YLLs) also increased from 2,097,154 in 1990 (95% CI: 1,699,743-2,551,170) to 3,580,692 in 2021 (95% CI: 2,826,228-4,518,921), though the age-standardized rate slightly decreased, from 44.175 per 100,000 to 41.791 per 100,000 (AAPC = -0.3503, 95% CI: -0.4215 to -0.2791,*p*=0). In China, YLDs increased from 37,327 in 1990 (95% CI: 24,503-51,465) to 94,133 in 2021 (95% CI: 61,403-131,967), with the age-standardized rate rising from 3.64 per 100,000 to 4.549 per 100,000 (AAPC = 0.8037, 95% CI: 0.4677 to 1.1407,*p*=0.000003). Meanwhile, YLLs in China decreased from 879,426 in 1990 (95% CI: 700,619-1,076,146) to 791,891 in 2021 (95% CI: 591,272-1,031,085), with the age-standardized rate declining from 77.275 per 100,000 to 43.404 per 100,000 (AAPC = -1.9475, 95% CI: -2.1372 to -1.7574,*p*=0). This suggests that, while quality of life has worsened for both global and Chinese populations, China has made notable progress in reducing premature deaths due to CKD.

### 3.2 Trends in the burden of chronic kidney disease due to type 1 diabetes in China and globally

Both China and the global population have experienced significant changes in the burden of CKD due to type 1 diabetes over the past few decades, with notable differences in trends for incidence, prevalence, mortality, and disease burden indicators.

**Figure 1:**
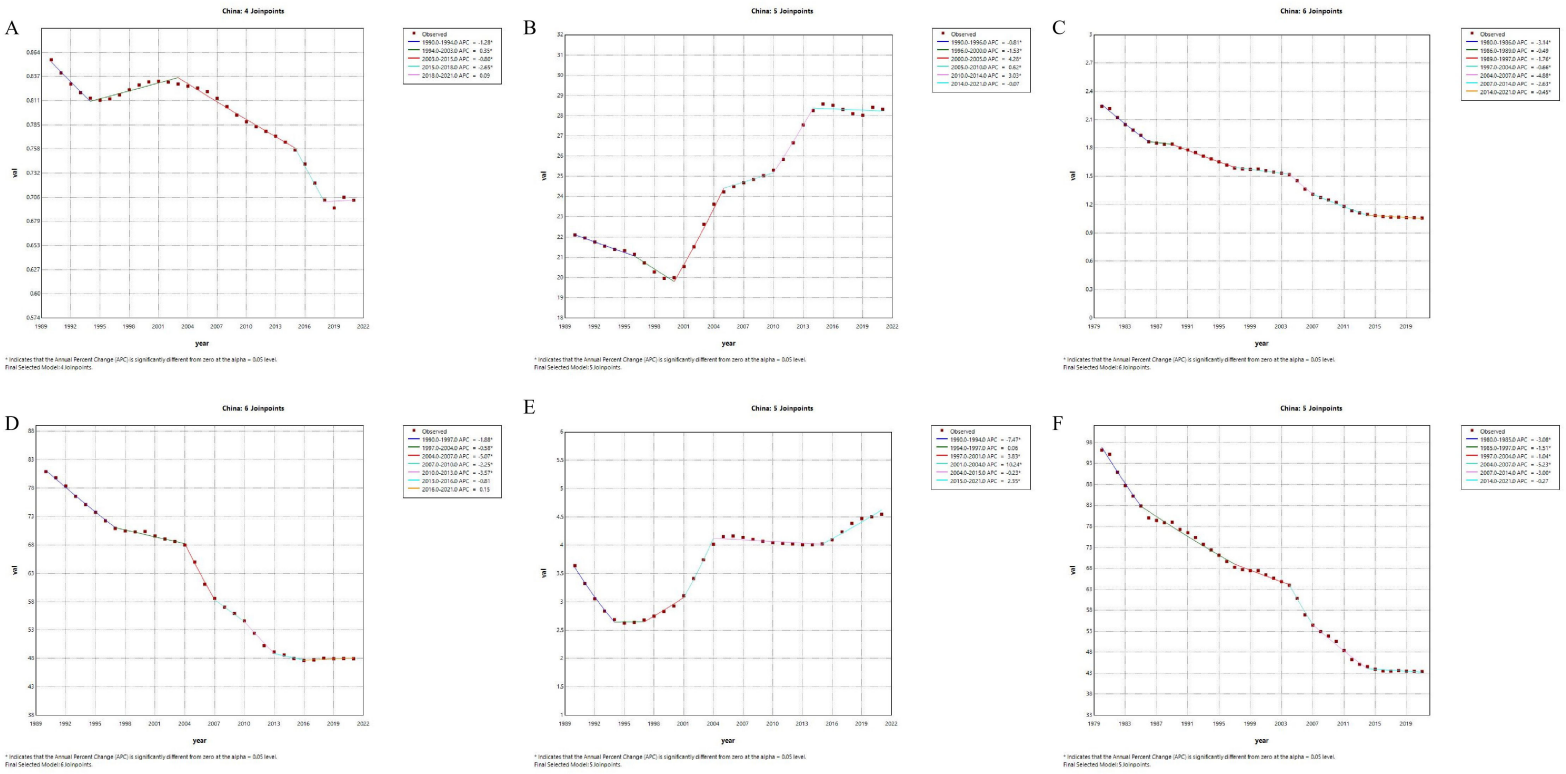
Annual Percentage Change (APC) trends of incidence, prevalence, mortality, disability-adjusted life years, years lived with disability, and years of life lost due to type 1 diabetes-related chronic kidney disease in China from 1990 to 2021. In China, the incidence increased slowly from 1990 to 2005 and then surged significantly, stabilizing in recent years **(A)**. Prevalence has been continuously increasing, with a marked acceleration after 2000 **(B)**. Mortality shows a consistent decline **(C)**. The DALYs rate declined from 1990 to 2000, but increased significantly after 2011 **(D)**. YLDs decreased before 2000 and have been rising continuously since **(E)**. YLLs showed an initial decline, then leveled off **(F)**. Solid points represent actual observed data, while the dashed line represents the APC model trend derived from Joinpoint regression analysis. Different color segments indicate trends in different time periods, along with their corresponding APC values, and asterisks (*) denote periods with significantly different APC values (p < 0.05).

**Figure 2:**
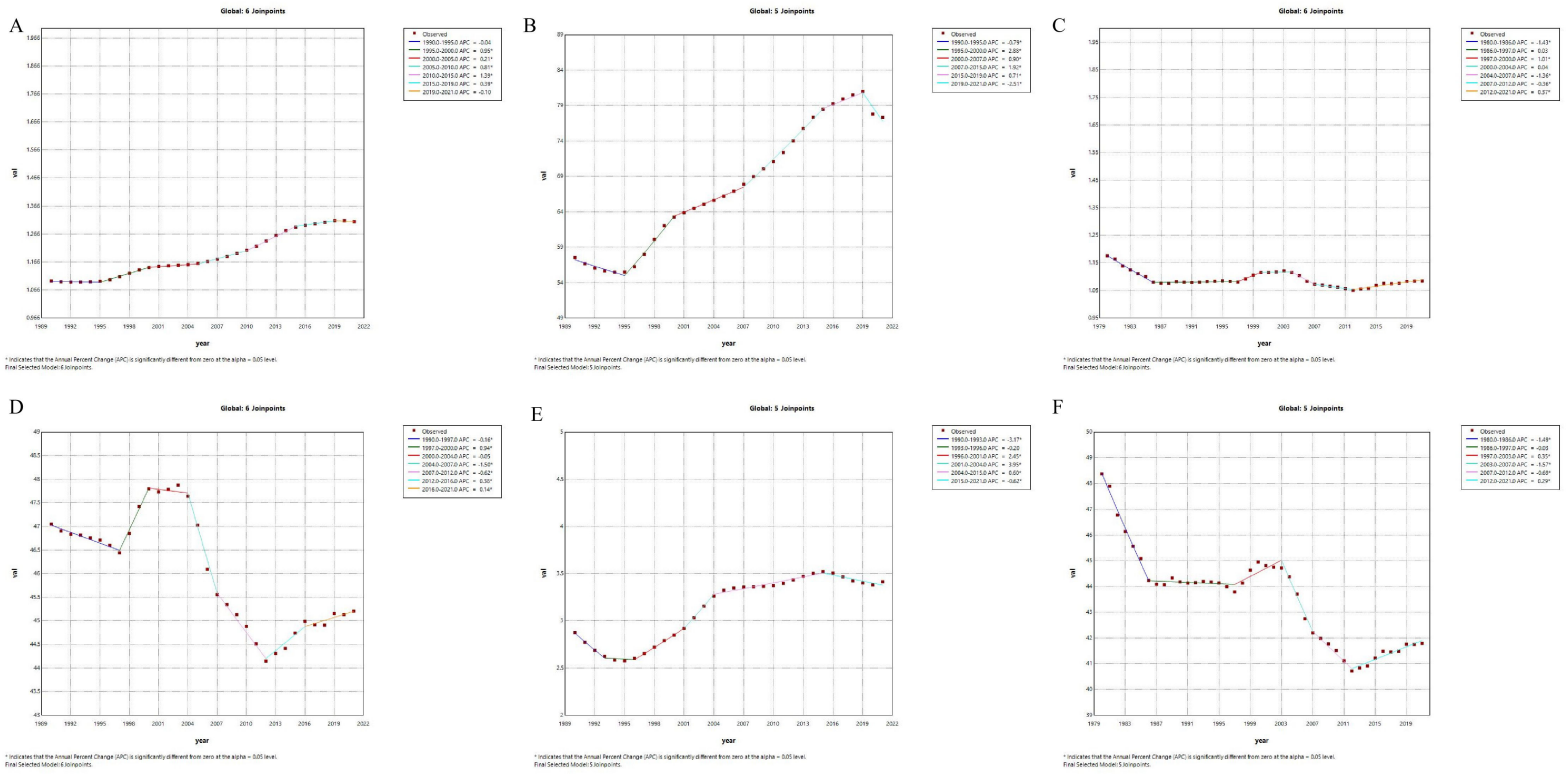
Annual Percentage Change (APC) trends of incidence, prevalence, mortality, disability-adjusted life years, years lived with disability, and years of life lostdue to type 1 diabetes-related chronic kidney disease globally from 1990 to 2021. Globally, the incidence has been increasing, especially after 2000, with recent stabilization **(A)**. Prevalence continues to rise, with a particularly rapid increase from 1990 to 2005, followed by a slower pace **(B)**. Mortality has been generally declining **(C)**. The DALYs rate decreased significantly from 1990 to 2000, then fluctuated and increased thereafter **(D)**. YLDs rate grew slowly before 2000, then accelerated significantly **(E)**. YLLs rate decreased rapidly from 1990 to 2000, and has since leveled off **(F)**. Solid points represent observed data, while the dashed line represents the APC model trend derived from Joinpoint regression analysis. Different color segments indicate trends in different time periods, along with their corresponding APC values, and asterisks (*) denote periods with significantly different APC values (p < 0.05).

First, in terms of incidence, China has generally shown a downward trend since 1990, particularly with an accelerated decrease between 2010 and 2019, where the annual rate of change was -2.5% (**Figure 1**). This decline suggests effective public health strategies and improved management of type 1 diabetes, contributing to fewer new cases. Conversely, the global incidence has exhibited an upward trend since 1990, with a relatively rapid increase from 2005 to 2010 (APC = 0.81, *p*=0), followed by a slower increase after 2015 (APC = 1.39, *p*=0) (**Figure 2**). Secondly, the trends in prevalence have diverged. In China, the prevalence has significantly increased over the past several decades, with the most rapid growth occurring between 2000 and 2014 (APC = 4.28, *p*=0), followed by a slight slowdown after 2015 (APC = -0.07, *p=*0.418744). Globally, the prevalence also grew rapidly, with the highest increase between 1995 and 2019 (1995-2000:APC = 2.88, *p*=0, 2007-2015:APC=1.92, *p*=0), but has slightly declined since 2019 (APC = -2.51, *p=*0.000079). This indicates that China’s prevalence has shown a more sustained and significant increase compared to the global trend, suggesting that China faces greater challenges in long-term management and disease prevention.Regarding mortality and disease burden indicators (DALYs, YLDs, YLLs), both China and the global population have shown decreasing trends, but the specific patterns differ. China’s mortality rate has declined continuously since 1970, particularly experiencing the fastest decrease between 1990 and 2014 (2004-2007:APC = -4.88, *p*=0), whereas the global mortality rate has remained relatively stable, with a slight increase only between 1997 and 2000 (APC = 1.01, *p=*0.013265).For DALYs, China has shown a significant downward trend, especially after 2000, with a high rate of reduction between 2004 and 2013 (2004-2007:APC = -5.07, *p*=0; 2010-2013:APC=-3.57, *p*=0.000002), while the global DALYs decreased most rapidly between 2004 and 2012 (2004-2007:APC = -1.50, *p*=0.000001).After 2012, the global DALYs began to gradually increase.(2012-2016:APC = 0.38, *p=*0.000789). These trends suggest that while China has made sustained improvements in reducing disease burden, global efforts to reduce DALYs hatableve slowed in recent years, possibly due to increasing prevalence and aging populations. The global improvements in DALYs have primarily occurred in the earlier stages.In terms of YLDs, China experienced a significant increase between 1995 and 2010 (1997-2001:APC = 3.83, *p*=0.000003;2001-2004:APC=10.24, *p*=0), but the rate stabilized after 2004 (2004-2015:APC = -0.23, *p*=0.012057). Globally, YLDs increased rapidly from 1995 to 2004 (2001-2004:APC = 3.95, *p*=0.000305), then gradually slowed, with a slight decrease after 2016 (APC = -0.62, *p*=0.00056). Finally, in terms of YLLs, both China and the global population have experienced significant declines. China saw the most rapid reduction between 2004 and 2014 (2004-2007:APC = -5.23, *p*=0.000007;2007-2014:APC=-3.00, *p*=0), while the global decrease was greatest between 2003 and 2012 (2003-2007:APC = -1.57, *p*=0;2007-2012:APC=-0.69, *p*=0.000077). This decline reflects improvements in both life expectancy and disease management, but the pace of improvement has slowed down in both regions, particularly in the last decade. However, after 2012, there was an increasing trend in global YLLs (2012-2021:APC=0.29, *p*=0.000001), and the rate of decline in China also slowed down(2012-2021:APC=-0.27, *p*=0.147136).

### 3.3 Comparison of Trends and Regional Characteristics of the Burden of Chronic Kidney Disease Due to Type 1 Diabetes in China and Globally

**Figure 3:**
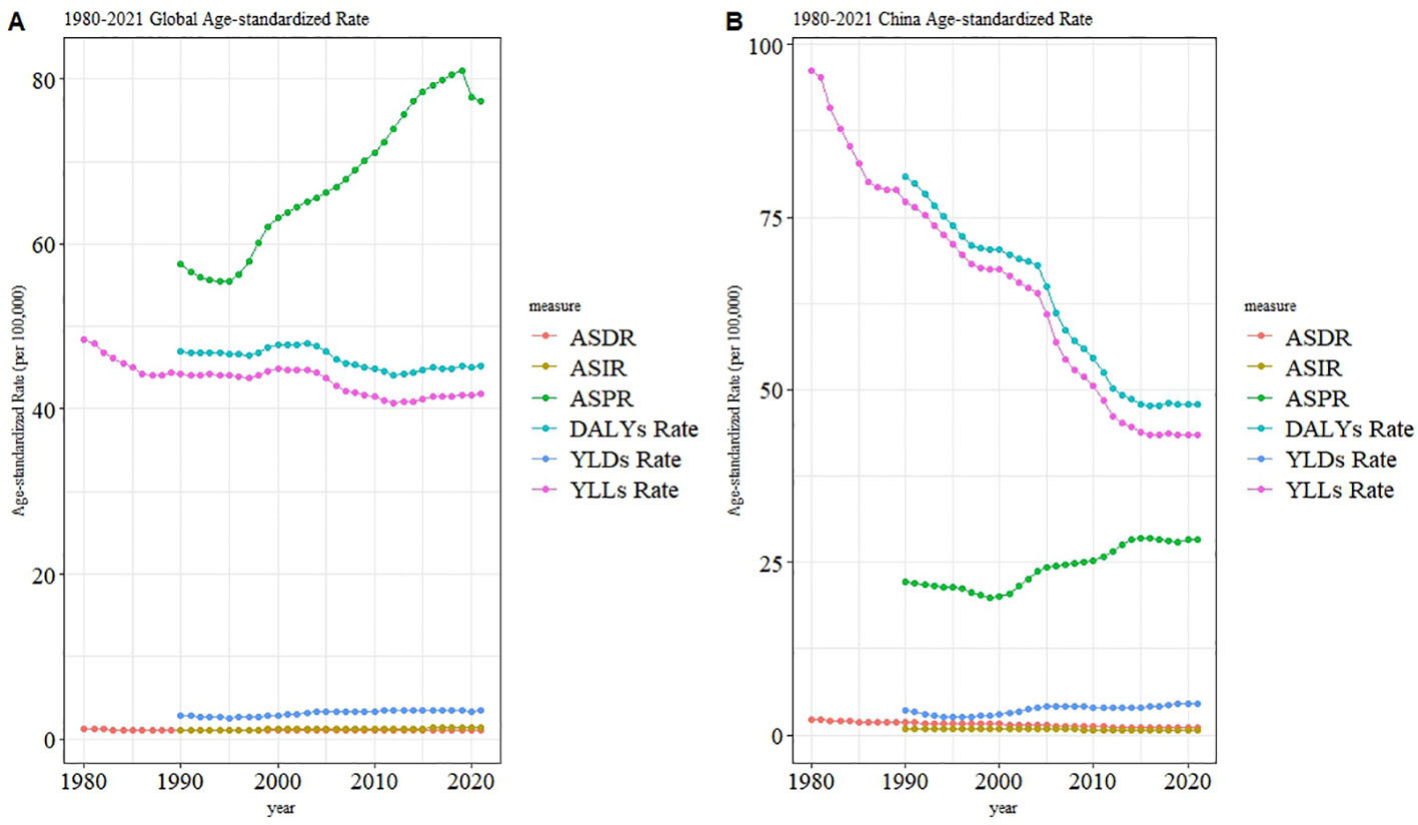
Age-standardized rates (ASR) of type 1 diabetes-related chronic kidney disease from 1980 to 2021 globally **(A)** and in China **(B)**, including age-standardized death rate (ASDR), incidence rate (ASIR), prevalence rate (ASPR), disability-adjusted life years (DALYs) rate, years lived with disability (YLDs) rate, and years of life lost (YLLs) rate. Globally **(A)**, ASPR has shown a significant increasing trend, and YLDs rate has also steadily risen, while ASDR and YLLs rate have generally remained stable or slightly declined. In China **(B)**, a notably different pattern is observed: ASPR grew rapidly after 2000, in line with the trend of YLDs, while ASDR and YLLs rates have significantly decreased since 1980.

Between 1990 and 2021, both global and Chinese populations have experienced significant changes in the burden of CKD due to type 1 diabetes across various indicators, including ASIR, ASPR, ASDR, and disease burden indicators (DALYs, YLDs, YLLs). While there are similarities between the trends in both regions, notable differences also emerge (**Figure 3**). Globally, the ASIR has remained stable overall, while the ASPR has increased significantly since 2000, reflecting an improvement in patient survival rates. Meanwhile, YLDs have gradually increased, suggesting an increase in years lived with disability,while YLLs and DALYs have remained relatively stable, indicating no major changes in the overall disease burden.In contrast, China’s ASIR has also remained stable, but the ASPR has shown a slow increase since 2000. Additionally, YLLs decreased significantly between 1990 and 2000 and have stabilized thereafter, while DALYs also rapidly declined during the same period and then stabilized, reflecting China’s no progress in reducing the overall disease burden. However, both China and the global population have seen an increase in YLDs, with China’s growth being relatively slower. This suggests that improving the quality of life remains a critical challenge for the future. Overall, China has made more substantial progress in reducing premature mortality and overall disease burden, while the global prevalence rate has shown a more pronounced increase.

### 3.4 Thirty-year comparative analysis of the burden of chronic kidney disease due to type 1 diabetes in China and globally from an age-stratified perspective

**Figure 4:**
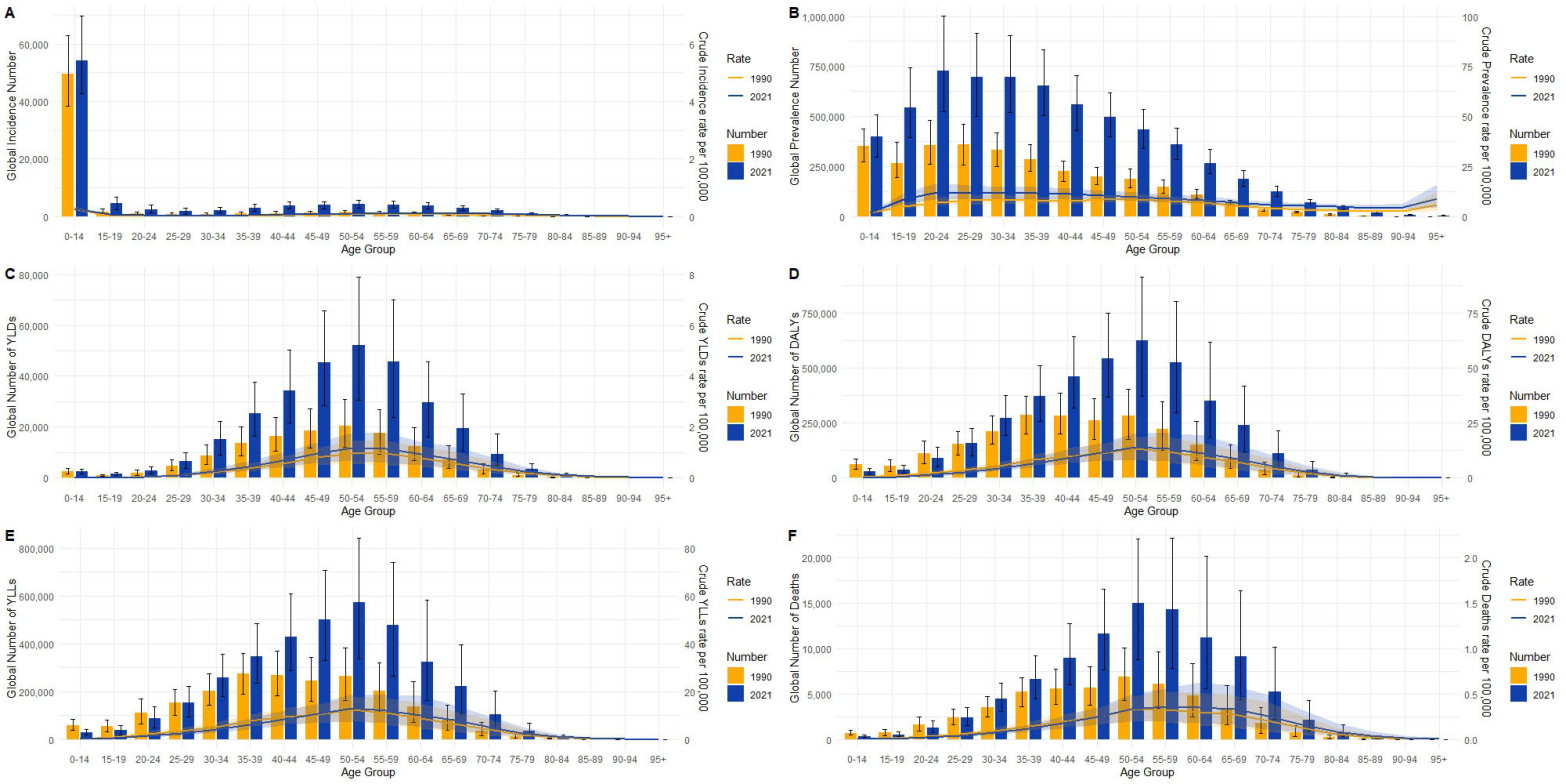
Distribution and crude rate changes of incidence **(A)**, prevalence **(B)**, years lived with disability (YLDs, **C**), disability-adjusted life years (DALYs, **D**), years of life lost (YLLs, **E**), and mortality **(F)** due to type 1 diabetes-related chronic kidney disease in different age groups globally in 1990 and 2021. Incidence **(A)** is notably higher in the 0-14 age group than in other age groups, with a slight increase in both incidence and crude incidence rate in 2021. Prevalence **(B)** increases progressively with age, particularly peaking in the 50-59 age group, with both prevalence and crude prevalence rates significantly higher in 2021 than in 1990. YLDs **(C)** peak in the 50-69 age group, and the crude YLDs rate in 2021 is much higher than in 1990. DALYs **(D)** also peak in the 50-69 age group, with a significant increase in crude DALYs rate in 2021. YLLs **(E)** peak in the middle-aged groups (50-69 years), with the crude YLLs rate declining in 2021 compared to 1990, reflecting an improvement in global mortality burden. Mortality **(F)** is concentrated in the middle-aged and older groups, especially those aged 60 and above, with a significant increase in mortality numbers in 2021, but a decrease in crude mortality rates compared to 1990.

**Figure 5:**
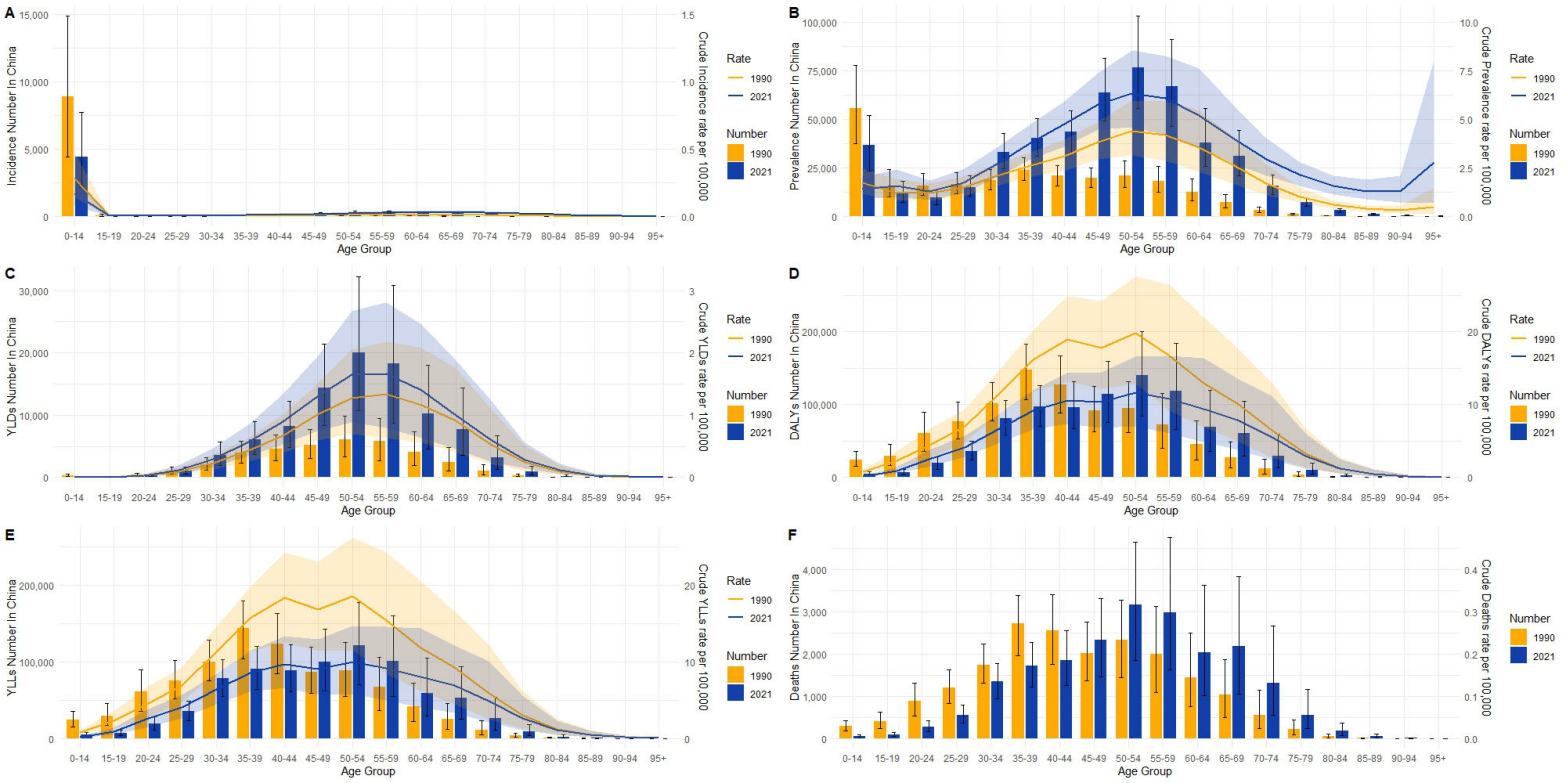
Distribution and crude rate changes of incidence **(A)**, prevalence **(B)**, years lived with disability (YLDs, **C**), disability-adjusted life years (DALYs, **D**), years of life lost (YLLs, **E**), and mortality **(F)** due to type 1 diabetes-related chronic kidney disease in different age groups in China in 1990 and 2021. The incidence is significantly higher in the 0-14 age group compared to other groups, with the crude incidence slightly higher in 2021 than in 1990. Prevalence increases significantly with age, peaking in the 50-59 age group, and both prevalence numbers and crude prevalence rates are significantly higher in all age groups in 2021 compared to 1990. YLDs and DALYs reach their peaks in the middle-aged groups (50-69 years), with the crude YLDs and DALYs rates significantly higher in 2021. YLLs and mortality are concentrated in the middle-aged and older age groups, especially those aged 60 and above, though crude YLLs and mortality rates in 2021 have decreased compared to 1990, indicating a reduction in the mortality burden.

From 1990 to 2021, the burden of CKD due to type 1 diabetes has exhibited significant changes in both global and Chinese populations across different age groups and various indicators. Globally (**Figure 4**), both the incidence and incidence rate (**Figure 4A**) were primarily concentrated in the 0-14 age group, with a decrease in both incidence and incidence rate in 2021 compared to 1990, suggesting some progress in the management of childhood diabetes worldwide. In contrast, China’s incidence rate also concentrated in the 0-14 age group but showed a more pronounced decline (**Figure 5**), indicating more effective early diabetes interventions and a successful reduction in new pediatric cases.

Regarding prevalence, globally, the prevalence rate and number of cases peaked in the 15-59 age group, with the prevalence rate in 2021 significantly higher than in 1990, indicating the continued expansion of the global CKD population (**Figure 4B**). In China, a similar trend was observed, although the rate of increase was relatively smaller (**Figure 5B**).

Among disease burden indicators, both global and Chinese data exhibited notable age-dependent variations, though with differing trends. YLDs showed that the burden in both regions was primarily concentrated in the 50-59 age group, with increases in YLDs in 2021 compared to 1990 (**Figures 4C**, **5C**). This suggests that, despite improvements in survival rates, the quality of life for patients has not been sufficiently improved in either global or Chinese contexts. Furthermore, DALYs reached their peak in the labor force age group (50–59) in both regions, with the absolute number and rate differing in 2021 compared to 1990. Globally, the increase in DALYs mainly reflects the ongoing rise in prevalence (**Figure 4D**), while in China, DALYs, although peaking in the same age group, showed a decreasing trend compared to 1990 (**Figure 5D**), indicating significant progress in reducing the overall disease burden, particularly through improvements in mortality rates and quality of life. Changes in YLLs further emphasize this trend. Globally, YLLs peaked in the 50-59 and 60-64 age groups but showed significant declines in both absolute numbers and rates by 2021, reflecting a marked reduction in the disease burden due to premature death. In China, this decline in YLLs was even more pronounced, particularly in the 50+ age group, highlighting China’s success in reducing premature mortality risks. However, in terms of death numbers (**Figures 4F**, **5F**), both China and the global population exhibited patterns linked to aging, with significant increases in deaths in the middle-aged and elderly groups (50+). Although mortality rates have decreased in both regions, the number of diabetes-related deaths in middle-aged and elderly populations continues to rise, emphasizing the need for continued attention to older populations.

### 3.5 Gender and age-stratified comparison of the burden of chronic kidney disease due to type 1 diabetes in China and globally

**Figure 6:**
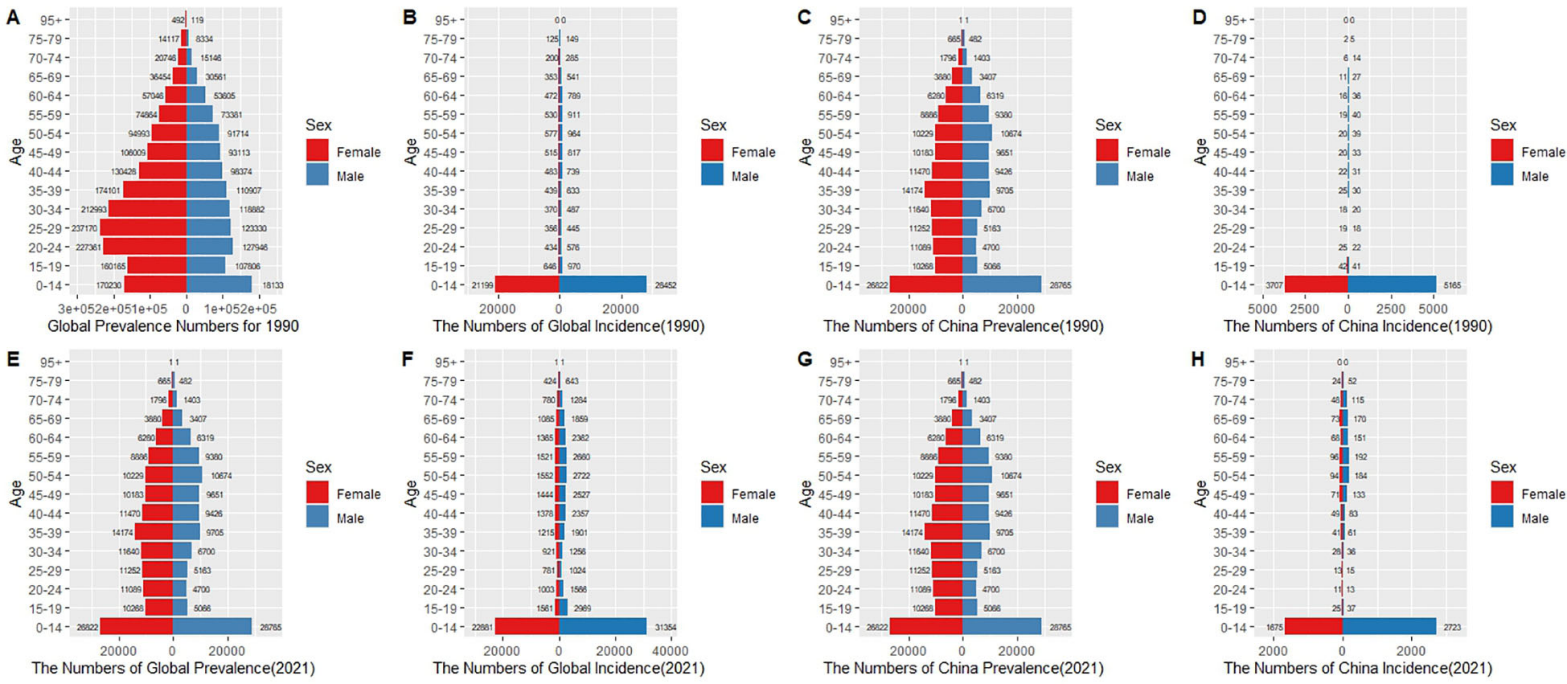
Comparison of prevalence (Prevalence Numbers, **A, C, E, G**) and incidence (Incidence Numbers, **B, D, F, H**) due to type 1 diabetes-related chronic kidney disease by sex (red for females, blue for males) and age distribution in China and globally in 1990 and 2021. In 1990, global prevalence **(A)** was highest in the 25-59 age group, with males slightly higher than females. Incidence **(B)** was most prominent in the 0-14 age group, with males significantly higher than females. In China, the prevalence **(C)** distribution was similar to the global pattern but with fewer cases, and incidence **(D)** also peaked in the 0-14 age group, with males clearly more affected than females. By 2021, global prevalence **(E)** continued to rise, with the 25-59 age group remaining the highest, and males still having slightly more cases than females. Global incidence **(F)** remained concentrated in the 0-14 age group, although the overall numbers decreased. In China, prevalence **(G)** showed a significant increase, with the 25-59 age group remaining the main affected group, while incidence **(H)** decreased in the 0-14 age group, although male incidence remained higher than female incidence.

Globally, in 1990 (**Figures 6A, B**), the number of cases for both males and females peaked in the 15-59 age group, with males in the 45-54 age group showing notably higher case numbers than females. This trend persisted in 2021 (**Figures 6E, F**), although the number of cases increased across all age groups, especially in the 45-64 age group, indicating that with improvements in healthcare, more patients are being diagnosed and surviving longer. In terms of incidence, in 1990 (**Figure 6B**), male incidence was primarily concentrated in the 0-14 age group, significantly higher than in females. By 2021 (**Figure 6F**), this age group’s incidence significantly decreased, though males still had higher incidence rates than females, indicating progress in childhood diabetes management worldwide, though gender disparities persist.

In China, in 1990 (**Figures 6C, D**), the number of cases in males was significantly higher than in females in the 15-59 age group, with the peak in the 50-54 age group. The incidence rate for children (0-14 years) was also significantly higher in males than in females. This trend continued in 2021 (**Figures 6G, H**), but unlike the global pattern, the increase in cases among the elderly (50+) in China, especially in the 55-74 age group, was more pronounced, reflecting the intensified impact of population aging on the disease burden. Moreover, the incidence rate in children under 14 showed a significant decline in 2021 compared to 1990, reflecting China’s achievements in diabetes screening and early intervention, although male children still showed a higher incidence than females, indicating persistent gender differences.

The changes in disease burden by gender and age in both China and globally exhibit some similarities and regional differences. Common trends include significantly higher case and incidence numbers in males across multiple age groups, especially in working-age groups (15–59), as well as a decline in childhood (0-14 years) incidence by 2021 compared to 1990. However, the global trend shows more significant increases in the 15-59 age group, whereas China’s increases are more concentrated in the elderly population (50+), reflecting the aging trend in China’s diabetes-related disease burden. Additionally, China’s decline in childhood incidence is more substantial, indicating the success of early prevention and intervention efforts, while the global decline is relatively smaller.

### 3.6 Forecast analysis of incident and prevalent case counts of chronic kidney disease due to type 1 diabetes in China and globally

**Figure 7:**
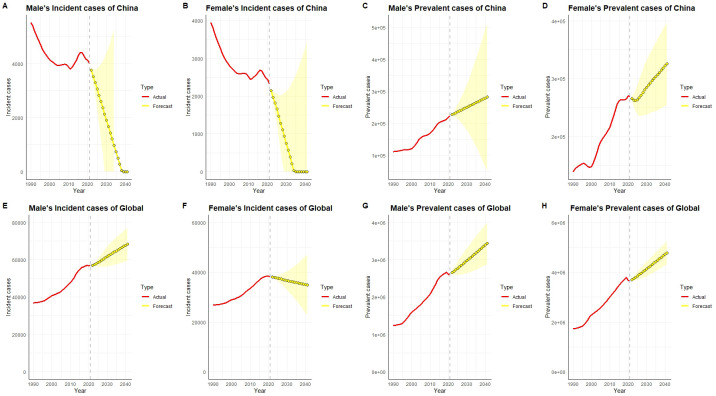
Historical data (actual values, red) and predicted trends (yellow) of the number of incident and prevalent cases of type 1 diabetes-related chronic kidney disease in males and females in China and globally from 1990 to 2040. In China, both male and female incident case counts **(A, B)** have shown a declining trend historically, especially after 2020. Predictions suggest that the number of incident cases in 2040 will further decrease to lower levels. However, the prevalent case counts **(C, D)** show a significant upward trend, particularly in females, with a more pronounced increase, and the trend is expected to continue. Globally, male and female incident case numbers **(E, F)** showed a continuous increase from 1990 to 2020, with males exhibiting a more significant rise. Predictions suggest that the global number of incident cases will remain high in 2040, with males still having a higher count than females. Globally, prevalent case counts **(G, H)** continue to increase, especially among females, with predictions indicating a further rise in 2040, reflecting a substantial increase in global disease burden.

In China, the number of incident cases has consistently decreased since 1990, with a particularly notable decline in females. Projections indicate that between 2022 and 2040, the incident case counts for both males and females in China will continue to decrease and approach an extremely low level, almost close to zero (**Figure 7**). However, the prevalent case counts show a significant upward trend, especially among females, indicating that despite the reduction in new cases, the total number of existing cases will continue to rise due to longer patient survival and cumulative effects.

In contrast, globally, the number of incident cases has remained relatively stable or slightly increased, which may reflect regional differences in diabetes prevention and control efforts. Projections suggest that the global number of incident cases will remain stable or show slight increases by 2040. Simultaneously, the number of prevalent cases has risen rapidly over the past 30 years and is expected to continue rising between 2022 and 2040, especially among females, with the growth rate in females significantly outpacing that in males. This trend reflects the prolonged survival of patients with type 1 diabetes globally and the cumulative increase in disease burden. China’s notable success in reducing incident case counts contrasts with the relative stability observed globally, but the overall upward trend in prevalence is consistent between China and the rest of the world.

## 4 Discussion

From 1990 to 2021, the burden of CKD caused by T1D showed different trends globally and in China, with significant differences observed across several aspects. On a global scale, the number of deaths due to T1D-related CKD increased year by year, from 49,300 cases in 1990 to 94,020 cases in 2021, indicating a gradual intensification of this disease burden. The epidemiology of type 1 diabetes among Arab children (<15 years) shows rising incidence rates, with significant regional variation influenced by environmental and nutritional factors (8). The global incidence of type 1 diabetes is increasing at a rate of 2% to 5% annually, with a prevalence of approximately 1 in 300 among individuals under 18 years old in the United States (21). However, the age-standardized mortality rate remained almost unchanged, suggesting that global efforts to control T1D-related CKD mortality have been relatively stable. Despite the increase in the number of deaths, the slight decline in the age-standardized mortality rate during this period reflects advancements in medical technology and treatment improvements (26–28). In contrast, while the number of deaths from T1D-related CKD in China also increased during this period, the age-standardized mortality rate dropped significantly from 1.8 per 100,000 to 1.058 per 100,000, highlighting substantial progress in reducing the mortality risk associated with this disease. The significant reduction in mortality in China may be closely linked to recent national policies promoting chronic disease management, treatment advancements, and improvements in medical resources (29–31).

Regarding the disease burden, global DALYs continued to increase, from 2,227,518 years in 1990 to 3,875,628 years in 2021. However, the age-standardized DALYs showed a slight decline, indicating that, although the overall burden has increased, the per capita burden has reduced. This trend reflects global progress in extending patient survival, but it also suggests the cumulative burden of chronic diseases due to prolonged survival (32–34). In China, DALYs associated with T1D-related CKD exhibited a decreasing trend, from 916,754 years in 1990 to 886,025 years in 2021. The age-standardized DALYs decreased substantially, demonstrating significant progress in reducing the disease burden caused by T1D-related CKD. China has made remarkable strides in alleviating the overall burden of chronic kidney disease, especially in terms of reducing premature deaths and improving patients’ quality of life through effective national health policies and the widespread adoption of early screening (35–38).

The prevalence of T1D-related CKD has been rising globally, with the number of cases increasing from 2,967,857 in 1990 to 6,295,711 in 2021, accompanied by a significant rise in the age-standardized prevalence. This trend reflects longer survival times and higher diagnosis rates among T1D patients. As the survival time of T1D patients has increased, the prevalence of CKD has also risen, indicating that the increasing disease burden is closely related to the extended patient lifespan (39, 40). This aligns with the increasing prevalence observed in many countries, where healthcare improvements have led to longer survival and better management of diabetes-related complications.In contrast, while the prevalence of T1D-related CKD has also increased in China, the growth has been relatively modest, and the trend in age-standardized prevalence has been more gradual. This may be attributed to China’s progress in early diabetes intervention and patient management (41, 42), as well as public health policies that have focused on managing diabetes complications and promoting early screening. Despite the increasing number of T1D patients in China, early screening and interventions have significantly improved disease control, effectively delaying disease progression.

Regarding incidence rates, the global trend has been gradually increasing. The global incidence increased from 63,601 cases in 1990 to 95,140 cases in 2021, with a continuous rise in the age-standardized incidence, indicating an increase in new cases and improvements in diagnostic capabilities (43, 44). This increase in incidence may also reflect greater awareness and access to healthcare, particularly in high-income regions, contributing to higher detection rates.In China, however, the incidence has shown a contrasting trend, continuously decreasing from 9,475 cases in 1990 to 6,321 cases in 2021, with a significant decline in age-standardized incidence. The downward trend in China’s incidence may be attributed to the country’s efforts in early identification, prevention, and management of T1D, particularly in the screening and management of children and adolescents (45, 46). This decline may also reflect the success of China’s public health interventions, including health education programs that focus on early detection and lifestyle modifications.

In terms of quality of life, there are also differences between global and Chinese trends. Globally, the YLDs due to T1D-related CKD have been rising steadily, from 130,365 years in 1990 to 294,936 years in 2021, indicating that, despite a decline in mortality, the quality of life for patients has not been significantly improved (47–49). This trend highlights the need for further improvement in the management of chronic kidney disease to not only extend survival but also improve patients’ quality of life. In contrast, while YLDs in China have also increased since 1990, the rise has been relatively smaller, suggesting that China has made more notable progress in improving patient quality of life. This may reflect improvements in healthcare infrastructure, patient management strategies, and access to treatment. Globally, the due to T1D-related CKD have increased, though the age-standardized rate has declined, reflecting improvements in medical technology and extended patient survival. In China, however, YLLs have shown a significant decrease, from 879,426 years in 1990 to 791,891 years in 2021, with a large reduction in the age-standardized rate, indicating substantial progress in reducing premature mortality.

From a gender and age perspective, the disease burden of T1D-related CKD in both global and Chinese populations exhibits some similarities and differences. Globally, the number of cases and incidences among males in the 15-59 age group is significantly higher than in females. This may be partially related to smoking and lifestyle factors (50–52). As medical technology improves, patient survival time increases, leading to a sustained rise in the burden of this group. In China, the number of cases in the 15-59 age group is also higher among males, particularly in the 50+ age group, where the burden of T1D-related CKD has significantly increased, reflecting the impact of an aging society on disease burden. Additionally, in both global and Chinese pediatric populations, the incidence has decreased, indicating that early intervention for T1D-related CKD has been effective in these groups, though the incidence remains significantly higher in male children than in females, highlighting the persistent gender disparity (53–55).

In terms of future projections for the burden of T1D-related CKD, both global and Chinese trends show some variation. In China, the ASIR has continued to decline, particularly among females, and it is projected that this trend will persist, approaching a near-zero level by 2040. This may be related to the decline in the younger population caused by China’s low fertility rate (56, 57). However, the ASPR is showing a marked upward trend, especially among females, suggesting that despite the reduction in new cases, the extended survival time of patients and the cumulative effects of chronic disease will continue to drive up the disease burden. In contrast, globally, the ASIR has remained relatively stable or slightly increased, reflecting differences in diabetes prevention and control across regions. The global increase in ASPR, particularly among females, underscores the global trend toward longer survival and accumulating disease burden among T1D patients. The global ASPR has increased rapidly over the past 30 years, and it is expected to continue to rise from 2022 to 2040, with a significant increase in females, indicating a global trend toward longer survival and accumulating disease burden among T1D patients (15, 58, 59).

Looking ahead, strategic intervention efforts will be crucial in managing the increasing burden of T1D-related CKD. Globally, improving early diagnosis rates, enhancing diabetes management, and strengthening healthcare infrastructure—particularly in low- and middle-income countries—are vital to alleviating the disease burden (60). Key strategies include increasing investments in diabetes education, preventive care, and ensuring affordable access to insulin therapy, which are essential for reducing the incidence of kidney-related complications (61). In China, the growing aging population underscores the need for focused health management strategies for the elderly and long-term care for T1D patients to mitigate the risk of chronic kidney disease (62). Strengthening healthcare systems to provide ongoing monitoring and personalized treatment plans, particularly for older individuals, will be paramount. Moreover, integrating diabetes and CKD management at the primary care level and promoting early screening will be important measures in alleviating the future burden (63). By addressing these challenges and capitalizing on opportunities for innovation in medical technology and health policy, both global and Chinese populations can mitigate the long-term impact of T1D-related CKD.

Overall, both global and Chinese trends reflect a similar growth in the disease burden of T1D-related CKD, although China has made significant progress in reducing mortality, improving quality of life, and mitigating disease burden, particularly in the management of pediatric diabetes and early intervention for chronic kidney disease. In the future, with advances in medical technology and further improvements in health policies, both global and Chinese populations may face distinct challenges and opportunities. Globally, improving early diagnosis rates and strengthening diabetes management, especially in low- and middle-income countries, will help alleviate the burden of T1D-related CKD. In China, as the aging population grows, greater attention should be paid to the health management of the elderly and long-term management of T1D patients to reduce the burden of chronic kidney disease.

### 4.1 Limitations

Despite the large sample size based on global and Chinese data, there are some limitations to this study. First, although data from the GBD database were used, differences in healthcare systems, health policies, and data collection methods across countries and regions limit the comparability of the data. Second, certain disease burden indicators (e.g., YLDs, YLLs) may be influenced by self-reporting, the level of health management, and uneven distribution of healthcare resources, which may not fully reflect the actual burden. Finally, this study did not delve into the pathophysiology and treatment outcomes of T1D-related CKD, which warrants further exploration through more targeted clinical studies.

## 5 Conclusion

The findings of this study indicate that the burden of chronic kidney disease due to type 1 diabetes continues to rise globally and in China, particularly with regard to prevalence and incidence. Although mortality rates and disease burden have improved globally, the overall burden continues to increase due to extended patient survival. China has made notable progress in reducing mortality and DALYs, indicating improvements in disease management. However, prevalence and incidence continue to rise, especially among children and adolescents, highlighting the need for early screening and intervention. Overall, while China has made progress in reducing the disease burden, the risk of increased burden from T1D-related chronic kidney disease remains, underscoring the need for strengthened early diagnosis and intervention efforts.

## Data Availability

Publicly available datasets were analyzed in this study. This data can be found here: https://vizhub.healthdata.org/gbd-results/.
