# Correction: Current status, trend changes, and future predictions of the disease burden of type 1 diabetes kidney disease in global and China

**DOI:** 10.3389/fendo.2025.1636897

**Published:** 2025-06-16

**Authors:** Qinghua Yang, Li Jin, Mingwei Luo, Shiwei Xie

**Affiliations:** ^1^ Department of Endocrinology, Panzhihua Central Hospital, Panzhihua, China; ^2^ Department of Medical Records Statistics, Panzhihua Central Hospital, Panzhihua, China; ^3^ Department of Orthopedics, Panzhihua Central Hospital, Panzhihua, China

**Keywords:** ARIMA prediction, chronic kidney disease, global disease burden, incidence, mortality, type 1 diabetes, trend analysis, years lived with disability

In the published article, there was an error in **Figure 7** and its legend as published. The original **Figure 7** was incorrectly labeled as showing ASIR and ASPR, while the actual data represent the total number of incident and prevalent cases (All ages + Number). Y-axis titles and subplot headings were also mislabeled and have now been corrected. The corrected **Figure 7** and its caption appear below.

**Figure 7:**
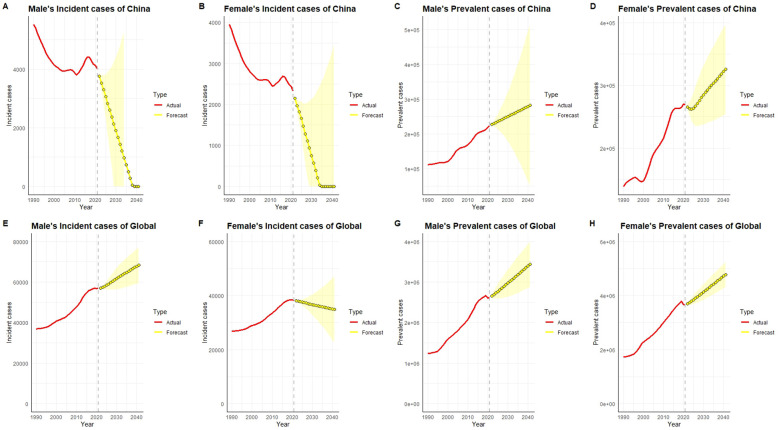
Historical data (actual values, red) and predicted trends (yellow) of the number of incident and prevalent cases of type 1 diabetes-related chronic kidney disease in males and females in China and globally from 1990 to 2040. In China, both male and female incident case counts **(A, B)** have shown a declining trend historically, especially after 2020. Predictions suggest that the number of incident cases in 2040 will further decrease to lower levels.However, the prevalent case counts **(C, D)** show a significant upward trend, particularly in females, with a more pronounced increase, and the trend is expected to continue.Globally, male and female incident case numbers **(E, F)** showed a continuous increase from 1990 to 2020, with males exhibiting a more significant rise. Predictions suggest that the global number of incident cases will remain high in 2040, with males still having a higher count than females.Globally, prevalent case counts **(G, H)** continue to increase, especially among females, with predictions indicating a further rise in 2040, reflecting a substantial increase in global disease burden.

In the published article, there was an error. The indicator names in "Section 3.6" were incorrectly stated as age-standardized incidence rate (ASIR) and age-standardized prevalence rate (ASPR), while the actual data used in the analysis and figure were based on the total number of incident and prevalent cases (All ages + Number).

A correction has been made to **Results**, *Section 3.6*.

The corrected paragraph appears below:

“3.6 Forecast analysis of incident and prevalent case counts of chronic kidney disease due to type 1 diabetes in China and globally

In China, the number of incident cases has consistently decreased since 1990, with a particularly notable decline in females. Projections indicate that between 2022 and 2040, the incident case counts for both males and females in China will continue to decrease and approach an extremely low level, almost close to zero (**Figure 7**). However, the prevalent case counts show a significant upward trend, especially among females, indicating that despite the reduction in new cases, the total number of existing cases will continue to rise due to longer patient survival and cumulative effects.

In contrast, globally, the number of incident cases has remained relatively stable or slightly increased, which may reflect regional differences in diabetes prevention and control efforts. Projections suggest that the global number of incident cases will remain stable or show slight increases by 2040. Simultaneously, the number of prevalent cases has risen rapidly over the past 30 years and is expected to continue rising between 2022 and 2040, especially among females, with the growth rate in females significantly outpacing that in males. This trend reflects the prolonged survival of patients with type 1 diabetes globally and the cumulative increase in disease burden. China’s notable success in reducing incident case counts contrasts with the relative stability observed globally, but the overall upward trend in prevalence is consistent between China and the rest of the world.”

The authors apologize for this error and state that this does not change the scientific conclusions of the article in any way. The original article has been updated.

